# Fly Ash Coated with Magnetic Materials: Improved Adsorbent for Cu (II) Removal from Wastewater

**DOI:** 10.3390/ma14010063

**Published:** 2020-12-25

**Authors:** Maria Harja, Gabriela Buema, Nicoleta Lupu, Horia Chiriac, Dumitru Daniel Herea, Gabriela Ciobanu

**Affiliations:** 1 Chemical Engineering Department, Faculty of Chemical Engineering and Environmental Protection, “Gheorghe Asachi” Technical University of Iasi, 73 Prof.dr.doc. Dimitrie Mangeron Street, 700050 Iasi, Romania; mharja@tuiasi.ro; 2 National Institute of Research and Development for Technical Physics, 47 Mangeron Boulevard, 700050 Iasi, Romania; nicole@phys-iasi.ro (N.L.); hchiriac@phys-iasi.ro (H.C.); dherea@phys-iasi.ro (D.D.H.)

**Keywords:** copper ions adsorption, Fe_3_O_4_, fly ash, isotherms, kinetic models, wastewater

## Abstract

Fly ash/magnetite material was used for the adsorption of copper ions from synthetic wastewater. The obtained material was characterized by scanning electron microscopy (SEM), energy dispersive X-ray analysis (EDAX), X-ray diffractometer (XRD), Fourier transform infrared spectroscopy (FTIR), Brunauer–Emmett–Teller (BET) surface area, and vibrating sample magnetometer (VSM). Batch adsorption experiments were employed in order to investigate the effects of adsorbent dose, initial Cu (II) concentration and contact time over adsorption efficiency. The experimental isotherms were modeled using Langmuir (four types of its linearization), Freundlich, Temkin, and Harkins–Jura isotherm models. The fits of the results are estimated according to the Langmuir isotherm, with a maximum adsorption capacity of 17.39 mg/g. The pseudo-second-order model was able to describe kinetic results. The data obtained throughout the study prove that this novel material represents a potential low-cost adsorbent for copper adsorption with improved adsorption capacity and magnetic separation capability compared with raw fly ash.

## 1. Introduction

The discharge of wastewater that contains heavy metals into aquatic environments is one of the most common sources of environmental pollution and is the reason why vital ecosystems are often affected [1,2].

Copper ions are one of the most widespread metals used in industry [3]. Among the three forms in which copper can be found, Cu^2+^ is considered to be the most toxic [4,5], leading to negative impacts on human health and the environment [6,7,8]. The allowable limit of copper ions in drinking water was established by World Health Organization at 2 mg/L. On the other hand, according to the United State Environmental Protection Agency (USEPA), the maximum copper concentration in industrial water is recommended to be 1.3 mg/L [9].

A variety of technologies have been applied for the treatment of waters contaminated with copper ions, among which the adsorption process is considered the most favorable alternative [10,11,12,13,14]. A series of materials were involved in Cu (II) adsorption, such as fly ash (FA) and modified fly ash [15,16], manganese ore [17], LSX zeolite [18], zeolite 4A [19], mesoporous silica [20], macro algae [21], and chitosan-based biodegradable composite [22]. Different methods have been developed for obtaining adsorbent materials, such as coprecipitation, chemical vapor depositions, plasma, electro-depositions, sol–gel, and ball milling. Of these methods, ball milling has the benefits of simplicity, low-cost, time-saving, no waste generation (water or solvent), ease of application, and possibility to be scaled up to the industrial level. [23].

Fly ash is preferred as an adsorbent since it is a cheap and highly-available material [24,25]. On the other hand, there are highlights that the presence of fly ash discharged from thermal power plants by the burning of coal represents a big environmental issue [26,27,28,29]. Unmodified fly ash presents small adsorption capacities. This fact could be explained through low surface area; hence, it is recommended to find a solution in order to remediate this problem. On the other hand, FA suspended in wastewater cannot be separated from the medium due to its too small particle size. Currently, the recycling of adsorbents is an actual issue [1] in the recovery process that is performed by centrifugation; unfortunately, this process exhibits high costs and energy consumption. A feasible alternative that would overcome these disadvantages consists of the use of magnetic adsorbents [30], with quick separation from wastewater [31].

Fe_3_O_4_ nanoparticles were utilized in a synthesis of various adsorbents due to some advantageous characteristics, such as the presence of surface functional groups, magnetic response ability, small particle size, biocompatibility, and biodegradability [32]. Thus, the obtaining of a new material based on the insertion of Fe_3_O_4_ and Fe_2_O_3_ within fly ash represents an interesting research field and a promising pathway to overcome these environmental problems.

The new material obtained by inserting Fe_3_O_4_ into fly ash particles can be used as adsorbent in wastewater treatment due to some advantages, such as its maximal number of active sites, its high surface area, and its high porosity. It is quickly separated from the solution by an external magnetic field without the use of supplementary steps such as filtration or centrifugation.

An important property to note is that the magnetic adsorbents, the core of which is a cluster of magnetic nanoparticles, do not show remanent magnetization. By removing the magnetic field, these adsorbents can be easily re-suspended in another solution. The adsorption–desorption processes can be performed due to this property. It should also be pointed out that secondary effluents are not generated [33,34].

The functional groups from the structure of unmodified fly ash would become active after combining with magnetite particles, thus yielding in a higher adsorption capacity of the synthesized materials. Also, the obtained material can be considered as a low-cost adsorbent.

The main objective of this paper was to obtain a low-cost and a very efficient magnetic adsorbent based on fly ash combined with Fe_3_O_4_ by a green method as well as to investigate the ability of the obtained composite to remove copper ions from synthetic wastewater. The effects of adsorbent dose, initial Cu (II) concentration, and contact time were checked. Furthermore, adsorption isotherms and kinetic models were investigated.

## 2. Materials and Methods

### 2.1. Materials

Class F fly ash was collected from a power plant operated by CET II Holboca, located in Iasi, North-East Romania. Generally, fly ash can contain many toxic trace elements that can be easily released into the environment. Leachability tests demonstrated that the FA used in this study did not contain any toxic trace elements such as Cr, As, Se, etc., and that it was not radioactive.

Fe_3_O_4_ was purchased from Alfa Aesar (Haverhill, MA, United States). All the chemical reagents were used as received.

Copper stock solution of 1000 mg/L was prepared by dissolving pentahydrate sulfate salt of copper in distilled water (Chemicals Company, Iasi, Romania). Thus, a quantity of 3.9294 g of CuSO_4_ 5H_2_O was dissolved in 1 L of distilled water in order to prepare 1000 mg/L of copper stock solution. The working solutions of 100–700 mg/L Cu (II) were obtained by diluting an exact volume of stock solution with distilled water. The 5-(4-nitrophenylazo) salicylic acid and 2,2′-dipyridyl solution (0.05%) were obtained by dissolving a quantity of the reagent in ethanol. The pH value of 3.5 necessary for metal ions complexation was obtained by using Citrate buffer solution [35].

Morphology of the adsorbent samples was observed with a field emission scanning electron microscope, JEOL JSM-6390 (Jeol USA Inc., Brno-Kohoutovice, Czech Republic). X-ray diffraction patterns were recorded using a Brucker AXS D8-Advance powder X-ray diffractometer with CuKa radiation, k = 0.1541 nm (Brucker, Brno, Czech Republic). Specific surface area was obtained with a Quantachrome instruments Nova 2200e model (Quantachrome Instruments, Graz, Austria). The magnetization data were acquired on a LakeShore 7410 vibrating sample magnetometer (VSM, Lake Shore Cryotronics, Inc, Westerville, OH, USA) in magnetic fields ranging between −20 and 20 kOe. Fourier transform infrared spectroscopy (FTIR) was performed on a Bruker Vertex 60 (Bruker Optik GmbH, Ettlingen, Germany) spectrometer.

### 2.2. Adsorbent Synthesis

The composite was obtained by milling 1 g of Fe_3_O_4_ with 9 g of raw fly ash (FA) in a planetary ball mill (PM-200 Retsch, Haan, Germany) for 4 h at 300 rpm. The grinding balls, 10 mm in diameter, and grinding bowls of the mill were made up of hard alloy tungsten carbide intended to operate in extreme conditions. The obtained magnetic material, noted as FA/Fe_3_O_4_, was washed with deionized water and dried at 60 °C for 24 h.

### 2.3. Adsorption Experiments

On the basis of previous researches on electroplating wastewater monitoring from Iasi area, the initial working concentration of synthetic solutions was established between 300–700 mg/L, the results being in accordance with the literature [36]. On the other hand, Al-Saydeh et al. (2017) states that copper is usually found at high concentrations in wastewater because it is the most used metal in various industrial applications, such as metal finishing, electroplating, plastics, and etching [9].

The equilibrium experiments were carried out at pH 5 using Berzelius beakers with 0.2 g adsorbent dispersed in 20 mL Cu (II) solution (initial concentrations of 100–700 mg/L).

The laboratory tests were carried out with intermittent stirring at room temperature. Cu (II) concentration in the supernatant was analyzed spectrophotometrically using 5-(4-nitrophenylazo) salicylic acid and 2,2′-dipyridyl in ethanol medium at 520 nm [37] with a Shimadzu UV-2450 DR UV–vis spectrophotometer (Shimadzu, Tokyo, Japan).

The adsorption study conditions are presented in Table 1.

The adsorption capacity, q (mg/g) and the adsorption efficiency, R (%), were calculated through Equations (1) and (2):

(1) q_e=(C_0−C_e)V/m

(2) R=(C_0−C_e)/C_0 ×100

where C_0 and C_e are the initial and equilibrium Cu (II) concentrations (mg/L), respectively, q is the amount of Cu (II) adsorbed onto FA/Fe_3_O_4_ (mg/g), V is the volume of Cu (II) solution (L), and m is the quantity of FA/Fe_3_O_4_ (g).

The adsorption capacity at different time intervals was calculated with Equation (3):

(3) q_t=(C_0−C_t)V/m

where C_t is Cu (II) concentration at different time intervals (mg/L), q_t is the amount of Cu (II) adsorbed onto FA/Fe_3_O_4_ at time intervals = 5–480 min, V is the volume of solution (L), and m is the quantity of FA/Fe_3_O_4_ (g).

## 3. Results

### 3.1. Characterization of FA/Fe_3_O_4_ Adsorbent

FA/Fe_3_O_4_ adsorbent was characterized through SEM, EDAX, XRD, FTIR, and VSM.

#### 3.1.1. SEM Analysis

**Figure 1:**
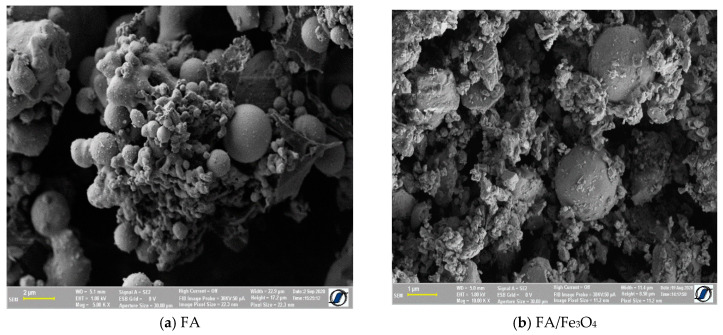

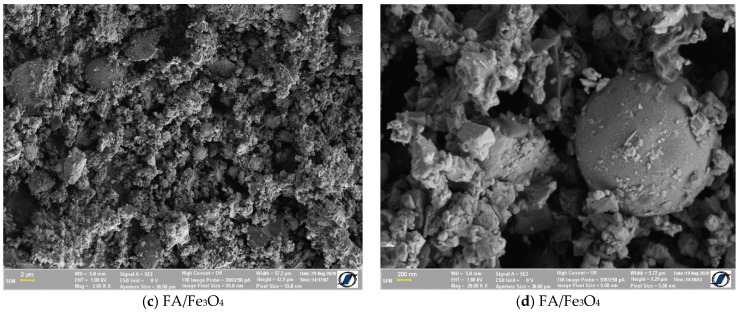
SEM image of the prepared composite.

The morphology shown in Figure 1 demonstrated that FA/Fe_3_O_4_ is composed by spherical particles, with large size distribution. Together with regular spherical particles, there were smaller irregular fly ash particles, which were likely derived from the high content of iron oxide and unburned carbon, as well as irregularly shaped amorphous particles. The sizes of the particles observed in Figure 1a are less than 5 µm, and the majority of the particles consisting in solid spheres ranged in size from 1 to 5 µm [38].

By milling, the agglomerated particles of FA were destroyed, and the magnetite added was uniformly distributed (Figure 1c). The SEM images show a uniform distribution of both small particle and magnetite within the composite. The SEM of the FA/Fe_3_O_4_ demonstrated that the fly ash was well crushed, and that the shapes of the particles became more uniform. The sizes were significantly reduced to about 600 nm, indicating the breakdown of the original spherical-shaped fly ash.

#### 3.1.2. EDAX Analysis

**Figure 2:**
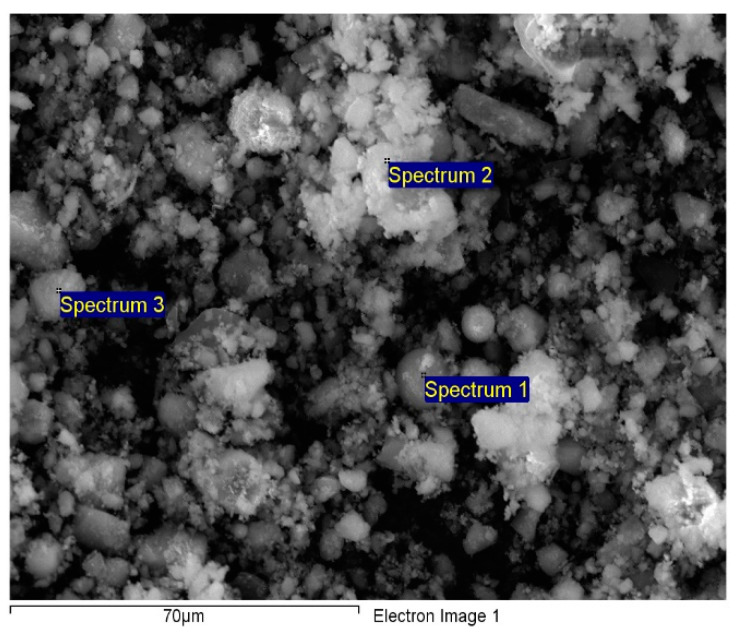

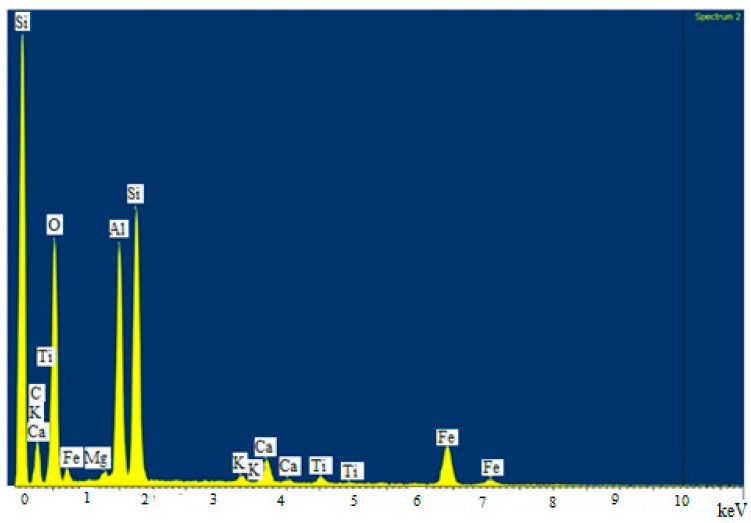
EDAX spectrum of FA/Fe_3_O_4_.

The chemical composition established through EDAX analysis is presented in Figure 2.

The comparison between raw material and synthesized material is presented in Table 2.

As determined by EDS, the predominant elements in the adsorbent samples in various compositions were oxygen, unburned carbon, silicon, aluminum, iron, and calcium [39]. Minor amounts of magnesium, titanium, sodium, and potassium were found in analyzed samples. By comparing the data obtained for Fe in the case of FA/Fe_3_O_4_ (7.74%) vs. FA (2.05%), it can be highlighted that the synthesis took place successfully. The mapping diagram demonstrated that the magnetite was uniformly distributed; consequently, milling time (4 h) led to a proper homogeneity.

#### 3.1.3. FTIR Analysis

**Figure 3:**
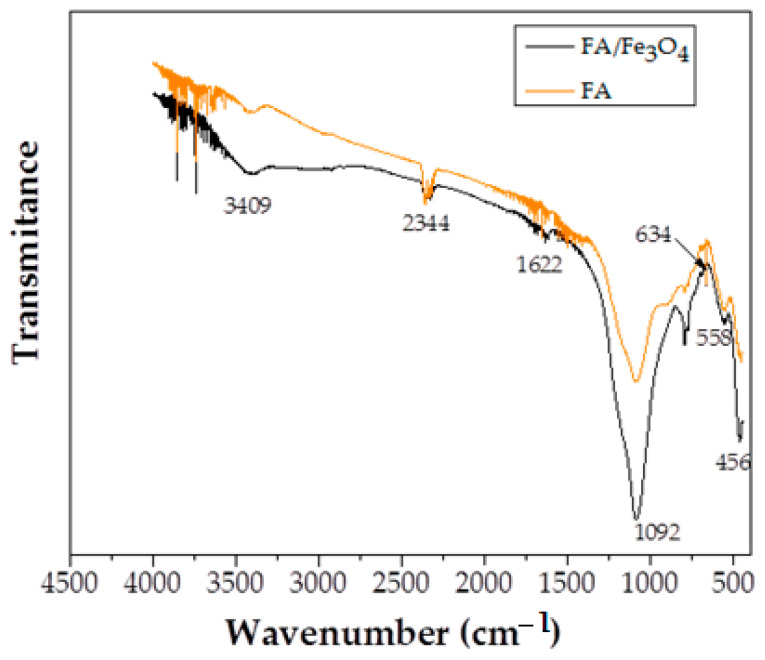
FTIR spectra of FA and FA/Fe_3_O_4._

The results of the FTIR analysis are illustrated in Figure 3. The FTIR analysis was realized to estimate the presence of the functional groups on the solid surface, the strength of the bonds, and the interactions between the surface functional groups and the adsorbed Cu (II).

The band, observed at the ∼530 spectrum of FA/Fe_3_O_4_, corresponds to the Fe-O/Fe-OH vibration of magnetite phase. The significant peak at 634 cm^−1^ is characteristic to magnetite. Apart from these, no significant change was observed between the FTIR spectra of FA and FA/Fe_3_O_4_.

The peak at 456 cm^−1^ was assigned to Si-O/Al-O in plane bending vibration and Si-O bending vibration, and the peak at 558 cm^−1^ was attributed to the Si-O vibration [40].

On the other hand, the peak at 1092 cm^−1^ was assigned to the asymmetric stretching of Si-O-Si. The very small peaks corresponded to H-O vibration (the samples were dried before analysis).

Taking into consideration that the only compositional difference between samples was the content of Fe_3_O_4_, no transformations were obvious in the structure of the material. The only difference in Figure 3 emerges from the higher Fe_3_O_4_ content, leading to an increase in the intensity of the spectrum associated with FA/Fe_3_O_4_.

#### 3.1.4. XRD Analysis

**Figure 4:**
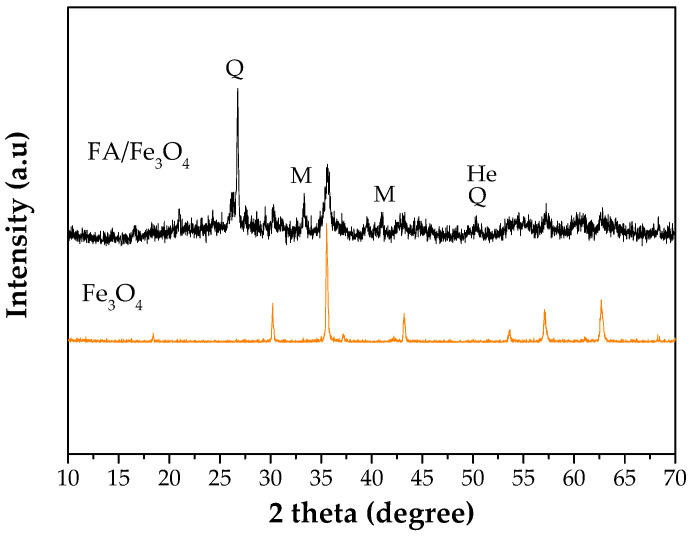
X-ray diffraction patterns of Fe_3_O_4_ and FA/Fe_3_O_4_.

The XRD analysis was performed in order to receive information about the mineralogical composition of the synthesized adsorbent, the results being presented in Figure 4. As it can be seen from the Figure 4, FA/Fe_3_O_4_ has the crystal phases of mullite (M) and quartz (Q). According to the X-ray pattern, the hematite (He) was found in the synthesized material. Also, from Figure 4 it can be noted that the XRD curve of FA/Fe_3_O_4_ has an amorphous structure [41] due to the formation of broad bands, and a crystalline phase within a wide scanning interval of 10–70°. Besides these peaks originating from the ash, the peaks at 2 theta (degrees) equaling 18.35°, 30.35°, 35.8°, 43.06°, 57.12°, and 62.73° correspond to Fe_3_O_4_. Additionally, an important variation in the peak intensity can be noticed from Figure 4. The comparison between the XRD diffraction patterns indicated the introduction of Fe_3_O_4_ on the FA structure.

#### 3.1.5. BET Analysis

**Figure 5:**
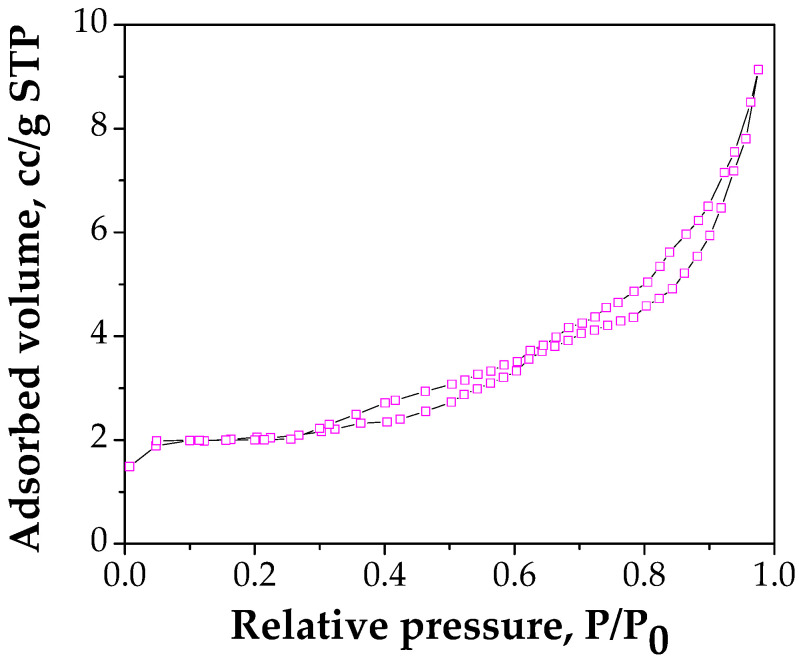
Nitrogen adsorption isotherm at 77 K on FA/Fe_3_O_4_.

The N_2_ adsorption–desorption isotherm for FA/Fe_3_O_4_ is shown in Figure 5.

The BET data shows that the specific surface area of FA/Fe_3_O_4_ is 6.153 m^2^/g, while the total pore volume is 0.0121 cm^3^/g. FA used in this study has the BET area of 4.03 m^2^/g and the total pore volume 0.009 cm^3^/g. The results show that the surface area of FA/Fe_3_O_4_ is 1.5 times higher compared with FA, which can be attributed to the interfacial interaction between FA and magnetite. Furthermore, FA/Fe_3_O_4_ is a mesoporous material, in accordance with the classification of IUPAC (International Union of Pure and Applied Chemistry, USA) [42], with an average pore volume of 7.85 nm.

#### 3.1.6. VSM Analysis

**Figure 6:**
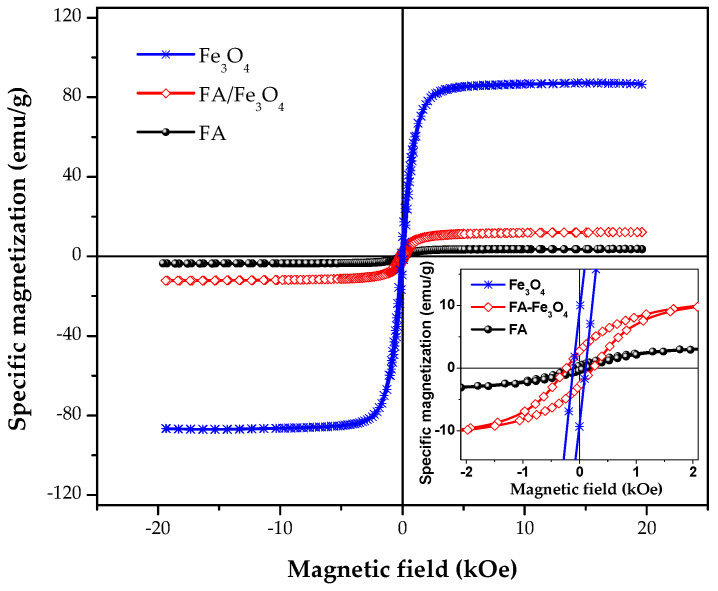
Magnetization hysteresis loop of the FA/Fe_3_O_4_ vs. FA.

The specific saturation magnetization (ssM) of the FA increased by more than 300% after mixing with Fe_3_O_4_ (Figure 6). Taking into account the mass ratio of 9:1 between FA and magnetite, a value of 88.6 emu/g can be calculated for the ssM of magnetite dispersed in the FA. This is consistent with the ssM obtained for magnetite, i.e., 87.1 (Figure 6), showing a negligible influence of the ball milling process on the magnetic properties.

From Figure 6, it can be observed that there are significant differences in the coercive field (Hc) and squareness values (Mr/Ms) of the samples. FA shows lower values for Hc and Mr/Ms ratio, being therefore less susceptible to agglomeration than FA-Fe_3_O_4_ particles. However, FA would need much more intense magnetic fields to be separated, after completing the water cleaning process, compared with FA/Fe_3_O_4_ particles.

### 3.2. Effect of Adsorption Parameters

#### 3.2.1. Effect of FA/Fe_3_O_4_ Dose

**Figure 7:**
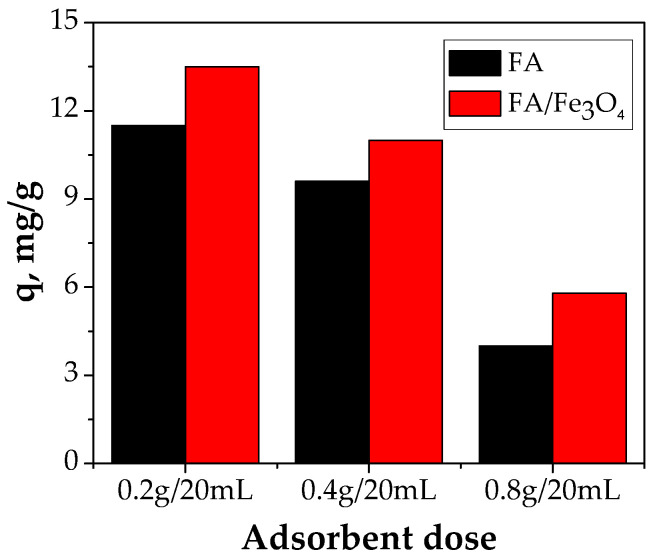
The effect of FA/Fe_3_O_4_ dose (experimental conditions—initial pH = 5.0; initial metal concentration = 300 mg/L; contact time = 24 h; temperature = 26 °C).

Generally, the adsorbent dose has a high impact on the adsorption capacity. To establish the effect of an FA/Fe_3_O_4_ dose on Cu (II) adsorption, a series of adsorption experiments were carried out using three adsorbent doses (0.2 g/20 mL, 0.4 g/20 mL, and 0.8 g/20 mL). The other parameters involved were an initial Cu (II) concentration of 300 mg/L, a pH of 5, a contact time of 24 h, and a temperature of 26 °C. It can be observed that with the increase in the FA/Fe_3_O_4_ dose, for a constant volume of solution and for the same initial concentration of Cu (II) ion, the adsorption capacity decreases. As shown in Figure 7, the best result was obtained using 10 g/L of FA/Fe_3_O_4_, with 13.48 mg/g of the Cu (II) adsorbed.

Also, FA presents the same trend. The adsorption capacity decreases with an increase in the adsorbent dose. The rationale behind this behavior might be related to the aggregation of the magnetic particles once the dose is increased, which consequently leads to the decrease in adsorption capacity. This fact was observed when the fly ash was treated with NaOH and H_2_SO_4_ [15,26]. Additionally, other researchers have obtained similar results [12,24].

#### 3.2.2. Effect of Initial Concentration

**Figure 8:**
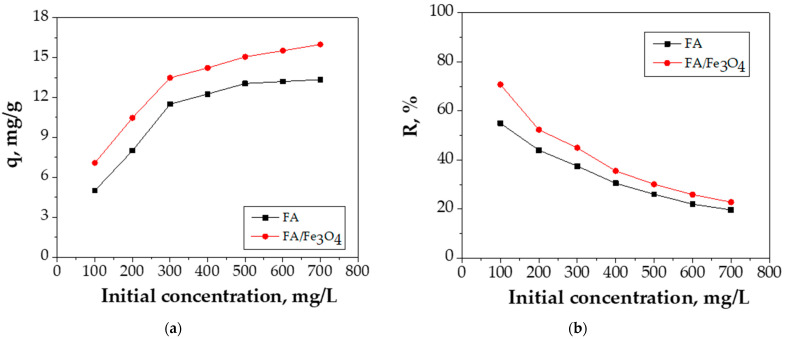
The effect of initial concentration on: (**a**) adsorption capacity, (**b**) adsorption efficiency (experimental conditions—initial pH = 5.0; adsorbent dosage = 0.2 g adsorbent/20 mL Cu (II) solution; contact time = 24 h; temperature = 26 °C).

The results regarding the influence of the concentration of FA and FA/Fe_3_O_4_ in the range of 100–700 mg/L are presented in Figure 8.

The following observations could be drawn from Figure 8: the lower Cu (II) concentrations (100 mg/L and 200 mg/L) show lower adsorption capacities; the initial Cu (II) concentration of 300 and 400 mg/L mark out the adsorption capacities of 11.9 mg/g and 12.26 mg/g for FA, respectively, whereas 13.48 mg/g and 14.22 mg/g mark out the adsorption capacities for FA/Fe_3_O_4_; the increase in concentration toward 500 mg/L led to an adsorption capacity of 13.05 mg/g for FA and 15.05 mg/g for FA/Fe_3_O_4_; at an initial Cu (II) concentration of 500, 600, and 700 mg/L, the adsorption capacity was approximately similar, although the removal efficiency decreased.

The adsorption efficiency shows a decreasing trend with the initial concentration of the Cu (II) ions from 100 to 700 mg/L. The adsorption sites are rapidly occupied at low Cu (II) concentration. As the initial concentration of Cu (II) increased, the majority of the accessible adsorption sites were no longer available, leading to a decrease of the removal efficiency.

#### 3.2.3. Effect of Contact Time

**Figure 9:**
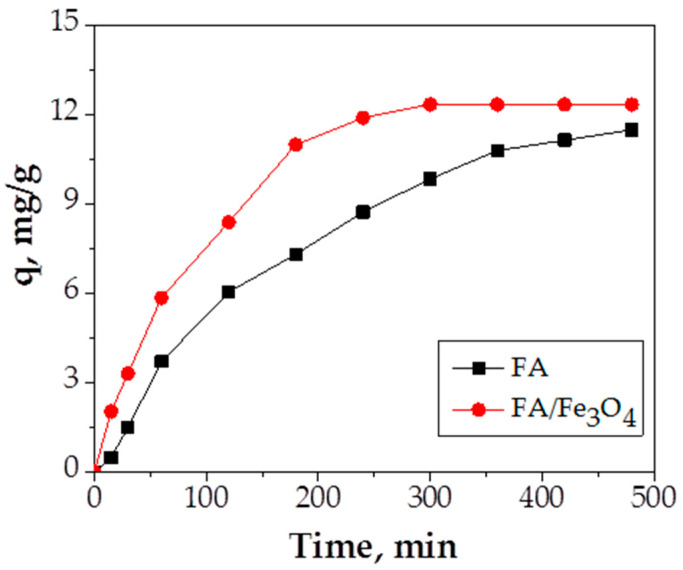
The effect of contact time (experimental conditions—initial pH = 5.0; adsorbent dosage = 0.2 g adsorbent/20 mL Cu (II) solution; initial metal concentration = 300 mg/L; temperature = 26 °C).

In order to establish the contact time necessary to reach equilibrium, different contact times (5–480 min) were used. The effect of contact time on Cu (II) adsorption capacity using FA and FA/Fe_3_O_4_ adsorbents is presented in Figure 9.

According to the obtained results, Figure 9 clearly proves that the contact time has an influence on Cu (II) adsorption capacity; by increasing contact time, the adsorption capacity increases. The results show that a maximum adsorption capacity of 12.21 mg/g is obtained in 4 h of contact in the case of Fe_3_O_4,_ as opposed to 11.9 mg/g after 6 h in the case of FA. The superiority of FA/Fe_2_O_3_ material was observed between 180 and 300 min of contact time. The reduction of contact time in the process of wastewater treatment saves energy and time. This fact shows that the insertion of Fe_3_O_4_ in the structure of raw fly ash represents a worthy advantage added to the induced magnetization.

### 3.3. Adsorption Isotherms

Adsorption isotherms and kinetic study offer valuable information regarding the adsorption process and specific properties of the adsorbent surface which are necessary for designing the adsorption systems.

**Figure 10:**
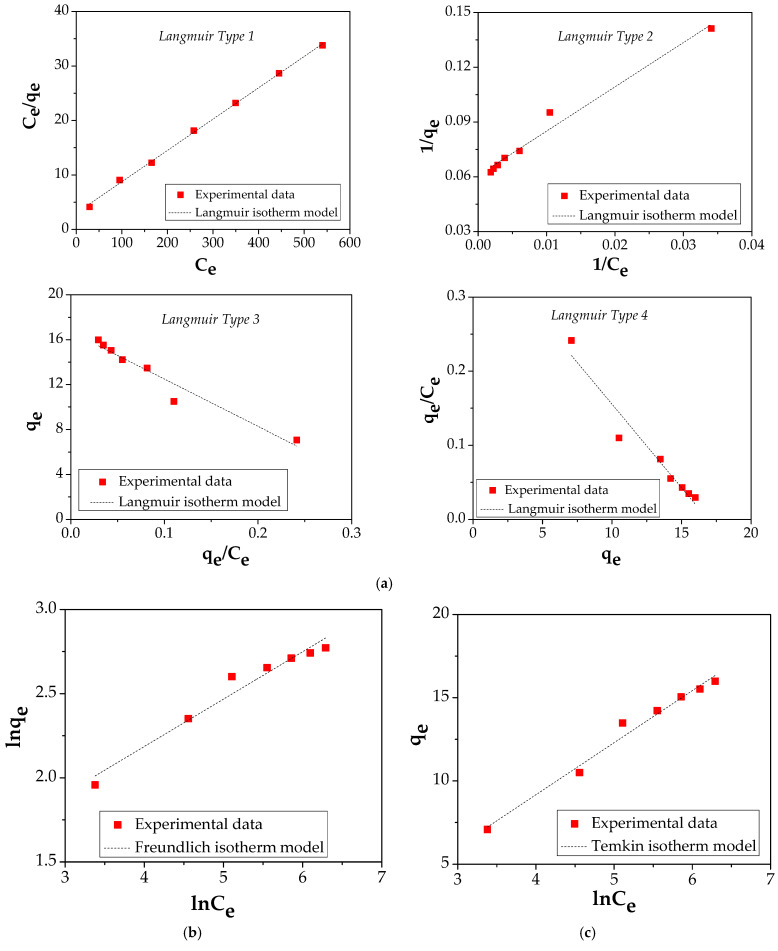

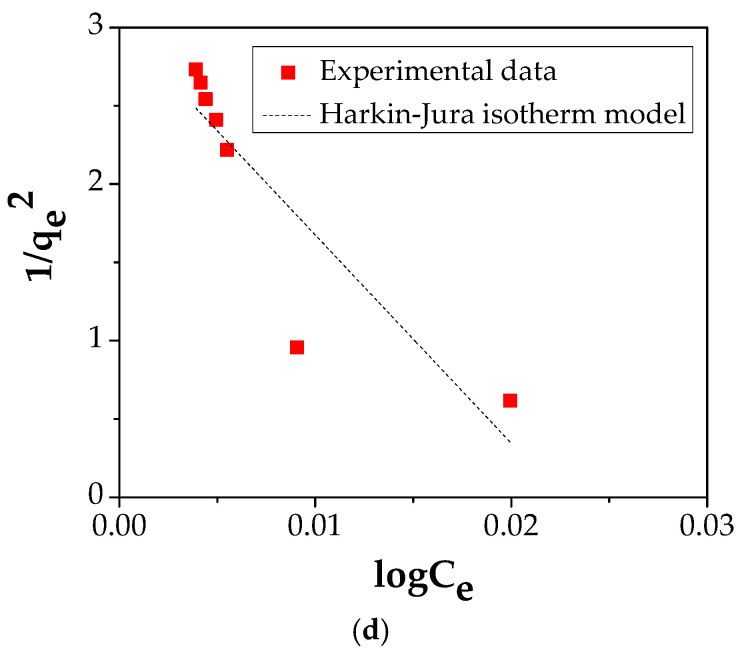
(**a**) Langmuir isotherm plot; (**b**) Freundlich isotherm plot; (**c**) Temkin isotherm plot; (**d**) Harkins–Jura isotherm plot.

The amount of Cu (II) adsorbed on the FA/Fe_3_O_4_ and the concentration of Cu (II) at equilibrium was explained using four common adsorption isotherms: Langmuir (four types of its linearization), Freundlich, Temkin, and Harkins–Jura (Figure 10 and Table 3). The related literature offers a complete description regarding the hypothesis, and the equation characteristic for each type of isotherm and kinetic model [20,43,44,45,46].

The correlated parameters of both equations are shown in Table 3. It should be mentioned that the value of experimentally obtained q_max_ is 15.991 mg/g.

In the case of FA/Fe_3_O_4_, by comparing the four isotherm models, it is noticed that the Langmuir equation shows a higher value of correlation coefficient, R^2^, compared with the Freundlich, Temkin, and Harkins–Jura isotherm models.

The four different linear Langmuir equations show that the adsorption capacities obtained are 17.39 mg/g for Type I, 16.44 mg/g for Type II, 16.71 mg/g for Type III, and 16.91 mg/g for Type IV, while the values of the  K_L are 0.0191, 0.025, 0.0237, and 0.0225 L/g, respectively. The value of the correlation coefficient, R^2^, of 0.9991, shows that Langmuir equation type 1 is able to describe the Cu (II) adsorption process onto FA/Fe_3_O_4_. Consequently, it can be concluded that the adsorption process is a monolayer uniform adsorption [47].

The nature of the adsorption process (favorable/unfavorable) is established according with the dimensionless separation factor, R_L_:

(4) R_L = 1/(1 + K_L×C_0)

where K_L is Langmuir constant and C_0 is the initial Cu (II) concentration in the range 100–700 mg/L.

**Figure 11:**
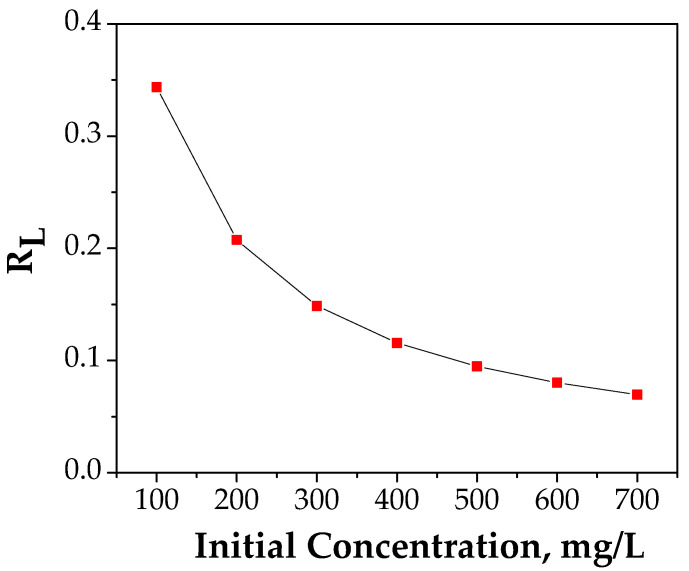
Plot of separation factor vs. initial Cu (II) concentration.

The fitting curve of R_L_ vs. C_0_ is presented in Figure 11.

The value achieved between 0 and 1 demonstrates that the adsorption process of Cu (II) onto FA/Fe_3_O_4_ is a favorable process [48].

**Figure 12:**
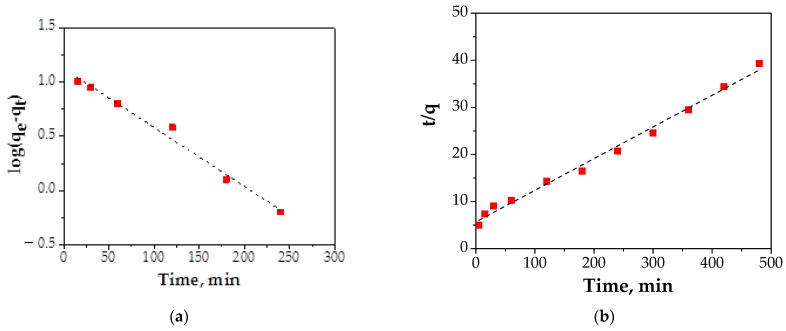
Kinetic adsorption plots: (**a**) pseudo-first-order model; (**b**) pseudo-second-order model.

The results obtained for two kinetic models: pseudo-first-order and pseudo-second-order are presented in Figure 12 and Table 4.

In the case of the pseudo-first-order equation, the value of correlation coefficient, R^2^, was 0.9871 with a reaction rate constant, k_1_, of 0.0124 (L/min). The k_2_ constant of the pseudo-second-order equation and R^2^ for Cu (II) adsorption onto FA/Fe_3_O_4_ were 0.00056 g/mg min and 0.993, respectively.

After applying the two kinetic models, it can be seen that the process of adsorption is described by the pseudo-second-order model. This fact indicated that the adsorption process of the Cu (II) ions onto FA/Fe_3_O_4_ was complex and more than one mechanism was involved [12]. Also, the parameter of the initial adsorption rate when t → 0 h was calculated using Equation (5).

(5) $$h\;={\;k}_{2}{q_{e}}^{2}$$

The value obtained was 0.0969 mg/g min.

These results suggested that the adsorption is predominantly chemical in nature.

Table 5 shows a comparison of the maximum adsorption capacities between the adsorbent prepared in this study and materials presented in the literature.

The FA/Fe_3_O_4_ material obtained in this study presents a comparable or even higher adsorption capacity in comparison with other materials used for Cu (II) adsorption.

This research represents a preliminary investigation. Further work will be focused on the optimization of the process related to the FA-Fe_3_O_4_ ratios and contact time, but also on the evaluation of the influence of ultrasonication applied at specific time points in order to disperse the particles aggregated during the adsorption process. The capacity of the magnetic composite to be magnetically separated from synthetic wastewater by using magnetic fields with different intensities and geometries will be also assessed.

## 4. Conclusions

An easy and simple method was used for the synthesis of a magnetic composite with fine adsorption properties. The effect of various variables, such as FA/Fe_3_O_4_ dose, initial Cu (II) concentration, and contact time were investigated. From the obtained results, it can be concluded that these three parameters have an important influence on copper adsorption capacity. The synthesized material can be successfully used in large domains of the initial concentration of Cu ions in the wastewater (100–700 mg/L).

The results demonstrated that for fly ash/magnetite material, the adsorption capacity increases with about 20% compared with FA. Also, an important thing to note is that the maximum adsorption capacity was reached in 4 h, while for FA the adsorption capacity attained the maximum value after 6 h. This emphasizes that the insertion of Fe_3_O_4_ represents a clear advantage. The adsorption capacities are higher compared with natural zeolites and close to those of zeolites synthesized from FA, but the proposed method is very easy and cheaper.

Not least, this novel material represents progress that is opposite of other low-cost adsorbents for copper removal since it can be quickly removed by magnetic separation.

Langmuir Type 1 isotherm can predict the experimental data with a maximum adsorption capacity of 17.39 mg/g. The Fe_3_O_4_ was inserted in the fly ash structure through the ball milling treatment and the material obtained did not break up into initial components.

Overall, it can be stated that this novel material represents a potentially low-cost adsorbent for copper removal, with improved adsorption capacity compared with the raw fly ash.

## Figures and Tables

**Table 1:** Adsorption study conditions.

Parameter Effect	
Dose of adsorbent → 0.2 g adsorbent/20 mL Cu (II) solution, 0.4 g adsorbent/20 mL Cu (II) solution, 0.8 g adsorbent/20 mL Cu (II) solution	Initial Cu (II) concentration: 300 mg/L; pH = 5
Initial Cu (II) concentration → 100 mg/L, 200 mg/L, 300 mg/L, 400 mg/L, 500 mg/L, 600 mg/L, 700 mg/L	Dose of adsorbent = 0.2 g adsorbent/20 mL Cu (II) solution; pH = 5
Contact time→ 5–480 min	Initial Cu (II) concentration: 300 mg/L; dose of adsorbent = 0.2 g adsorbent/20 mL Cu (II) solution; pH = 5

**Table 2:** Elemental analysis of FA and FA/Fe_3_O_4_, mass %.

Element	FA	FA/Fe_3_O_4_
C	18.27	18.25
O	45.82	46.72
Si	18.81	13.95
Al	11.09	10.22
Ca	1.75	1.7
Fe	2.05	7.74
K	0.79	0.41
Mg	0.60	0.34
Ti	0.74	0.67

**Table 3:** The correlated parameters of isotherms of Cu (II) adsorption onto FA/Fe_3_O_4._

Model	Parameter	Value
Langmuir type 1	q_max	17.39
	K_L	0.0191
	R^2^	**0.9987**
Langmuir type 2	q_max	16.44
	K_L	0.025
	R^2^	0.9792
Langmuir type 3	q_max	16.71
	K_L	0.0237
	R^2^	0.9487
Langmuir type 4	q_max	16.91
	K_L	0.0225
	R^2^	0.9487
Freundlich	K_F	2.88
	1/n	0.2816
	R^2^	0.9625
Temkin	B	3.1282
	b	0.792
	A_T_	3.25
	R^2^	0.9821
Harkins–Jura	A_HJ_	0.0075
	B_HJ_	0.0226
	R^2^	0.9002

where: q_max  is the maximum adsorption capacity (mg/g); K_L is Langmuir constant (L/g); K_F is the Freundlich constant; 1/n is the heterogeneity factor; A_T_ is Temkin isotherm equilibrium binding constant (L/g); b_T_ is Temkin isotherm constant; B is the constant related to heat of adsorption (J/mol); A_HJ_ and B_HJ_ are Harkins–Jura constants. The bold of 0.0987: to highlight the high value of R^2^.

**Table 4:** Kinetic parameters of Cu (II) adsorption onto FA/Fe_3_O_4_.

Kinetic Model	Parameters	Values
Pseudo-first order	k_1_, 1/min	0.0124
	R^2^	0.9871
Pseudo-second order	q_e_ cal, mg/g	15.64
	k_2_, g/mg min	0.00065
	R^2^	0.993

**Table 5:** Maximum Cu (II) adsorption capacities (q_max_).

Adsorbent	q_max_ (mg/g)	References
FA (Fly ash)	14.46	[15]
Fly ash treated with 5 M of NaOH at 90 °C, 4 h	27.904	[15]
PPy/Perlite (Polypyrrole composite on perlite zeolite)	3.57	[49]
ARH (Bentonite treated with sodium)	17.241	[50]
ARC (Bentonite treated with calcium)	18.181	[50]
ARS (Bentonite treated with sulphuric acid)	24.390	[50]
Modified clay	13–21	[51]
Natural zeolites	2.5	[14]
Fe_3_O_4_ particles with 1,6-hexadiamine	25.77–26.58	[52]
Magnetic Prussian blue	8.93	[53]
FA/Fe_3_O_4_	17.39	This study

## Data Availability

The data presented in this study are available on request from the corresponding author.
